# Multiple evanescent white dot syndrome associated with COVID-19 infection: a case report

**DOI:** 10.1186/s13256-024-04596-y

**Published:** 2024-06-08

**Authors:** Michael Ting, Jessica Hsueh, Jila Noori

**Affiliations:** 1 University of Oklahoma College of Medicine, Oklahoma City, OK USA; 2 Department of Ophthalmology, Dean McGee Eye Institute, University of Oklahoma Health Sciences Center, 608 Stanton L Young Blvd, Oklahoma City, OK 73104 USA

**Keywords:** Multiple evanescent white dot syndrome, COVID-19 infection, White dot syndrome, Retina, Ophthalmology, Case report

## Abstract

**Background:**

To report a case of Multiple Evanescent White Dot Syndrome (MEWDS) one month after a COVID-19 infection in a female patient at an age unusual for the occurrence of this disease.

**Case presentation:**

A 69-year-old Caucasian female reported the presence of floaters, photopsia, and enlarging vision loss in her left eye following the COVID-19 infection. Clinical and multimodal imaging was consistent with the MEWDS diagnosis. Fluorescein angiography examination revealed characteristic hyperfluorescent spots around the fovea in a wreath-like pattern. An extensive lab workup to rule out other autoimmune and infectious etiologies was inconclusive. Visual acuity and white dots resolved after a course of corticosteroids, which was confirmed on follow-up dilated fundus exam and multimodal imaging.

**Conclusions:**

MEWDS is a rare white dot syndrome that may occur following COVID-19 infection in addition to other reported ophthalmic disorders following this infection.

## Introduction

Multiple Evanescent White Dot Syndrome (MEWDS) is a rare, acute-onset inflammatory disease of the choroid and retina characterized by yellow-white dots at the level of the outer retina or retinal pigment epithelium. MEWDS typically affects young and middle-aged women and presents with unilaterally blurred vision, photopsia, floaters, and restricted visual field [1]. MEWDS has an excellent prognosis with complete recovery of vision and visual fields within a few months with rare recurrence. It develops after a viral prodrome around thirty percent of the time [2]. MEWDS pathogenesis is still unknown, but it is thought to be an ocular immune-mediated disorder in patients of specific genetic disposition. Vaccinations have also induced MEWDS in some rare cases, including the mRNA coronavirus disease 19 (COVID-19) vaccination in several case reports [3–7]. COVID-19 infection-related conjunctivitis, vision changes, irritation, and subconjunctival hemorrhage have been reported, but there are few reports of COVID-19 causing MEWDS [8]. Here, we report a case of MEWDS one month after a COVID-19 infection in a female patient at an age unusual for the occurrence of this disease.

## Case presentation

A 69-year-old Caucasian woman presented with new ocular symptoms in her left eye, which started about ten weeks before attending our uveitis clinic in March 2023. She was symptomatic with new floaters and central flashing, and about four weeks later, she developed an enlarging scotoma and progressive vision loss. The patient was diagnosed with a COVID-19 infection via antibody testing four weeks before the current onset of ocular symptoms, along with fever, congestion, and cough. The patient had previously received the COVID-19 vaccination several months before contracting COVID-19 infection. Aside from asthma, hypothyroidism, and headaches, her past medical history and family history were unremarkable and negative for prior autoimmune disease.

**Fig. 1:**
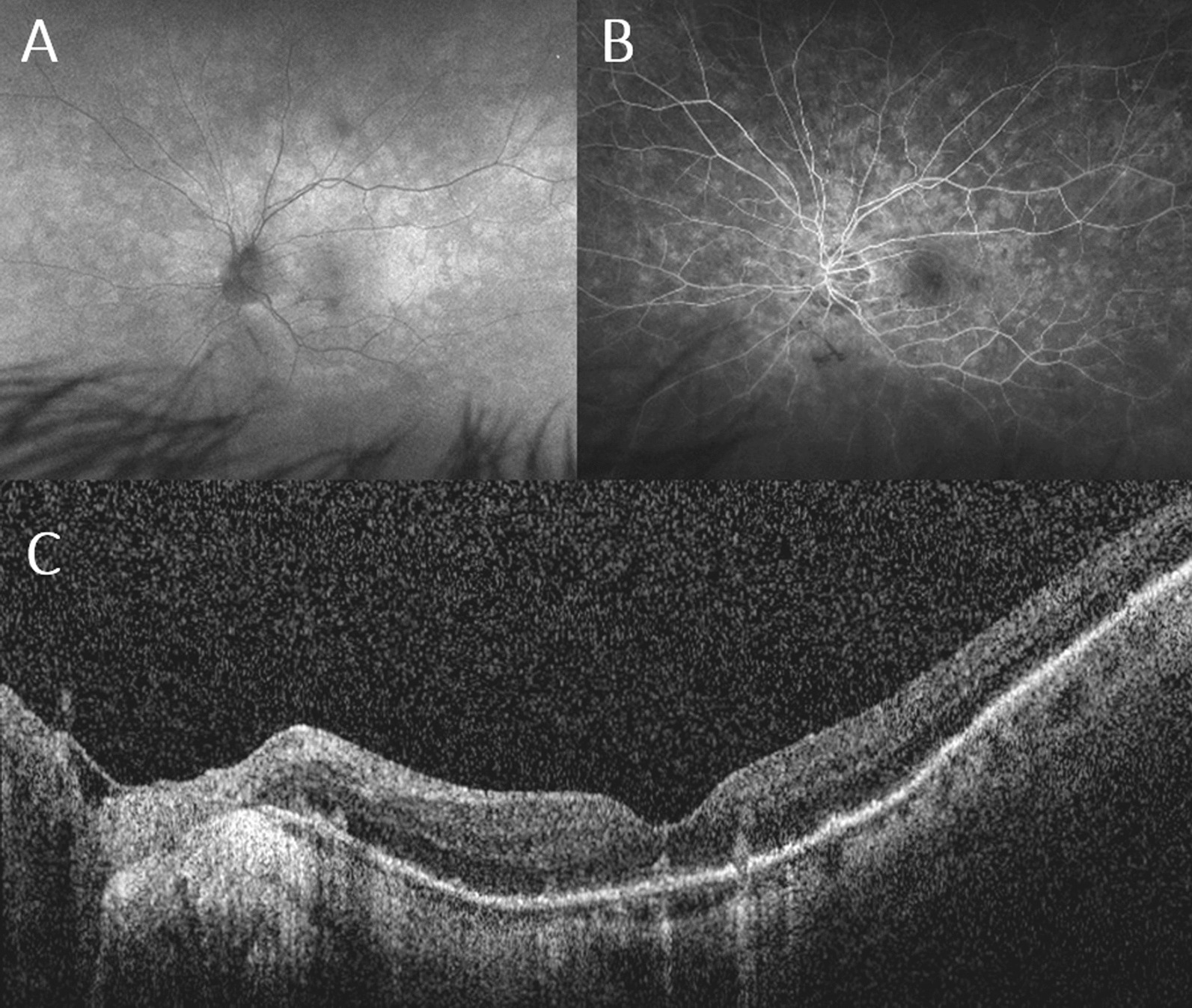
Multimodal imaging at the initial presentation. **A** Fundus autofluorescence (FAF) left eye at presentation demonstrating peripapillary and macular hyper autofluorescence extending to the equator. **B** Fluorescein angiography (FA) at presentation with hyperfluorescent in a wreath-like pattern corresponding to areas of hyper-autofluorescence on FAF. **C** Enhanced depth imaging optical coherence tomography (EDI OCT) at presentation showing hyperreflective subretinal deposits and significant ellipsoid zone disruption

A complete eye examination was performed with macular optical coherence tomography (OCT), fundus autofluorescence (FAF), and fluorescein angiography (FA) tests. ICG angiography was not done due to iodine allergy. The best corrected visual acuity was 20/30 in the right eye and 20/80 in the left eye. There was no relative afferent pupillary defect (RAPD). The ocular exam exhibited PVD with moderate vitreous inflammation and a quiet anterior chamber in the left eye. No sign of intraocular inflammation was detected in the right eye. The other ocular exam findings included bilateral mild nuclear cataract, myopic refractive error with mild myopic retinal degeneration. FAF showed peripapillary and macular hyper-autofluorescent spots in the posterior pole (Fig. 1A). These spots co-localized to punctate hyperfluorescent areas on FA, arranged in a wreath-like pattern with nearly well-defined borders (Fig. 1B). OCT examination featured disruption of the ellipsoid zone (EZ) and hyper-reflective subretinal deposits in the affected left eye (Fig. 1C).

Serum testing was conducted to rule out other possible etiologies. Complete blood count (CBC) and complete metabolic panel (CMP) were normal. QuantiFERON-TB Gold (QFT), angiotensin-converting enzyme (ACE) test, lysozyme blood test, HLA-B29 serotyping, antineutrophil cytoplasmic antibodies (ANCA), rapid plasma reagin (RPR) and fluorescent treponemal antibody absorption test (FTA-ABS) were all negative. C-reactive protein (CRP) and erythrocyte sedimentation rate (ESR) levels were slightly elevated compared to reference values.

**Fig. 2:**
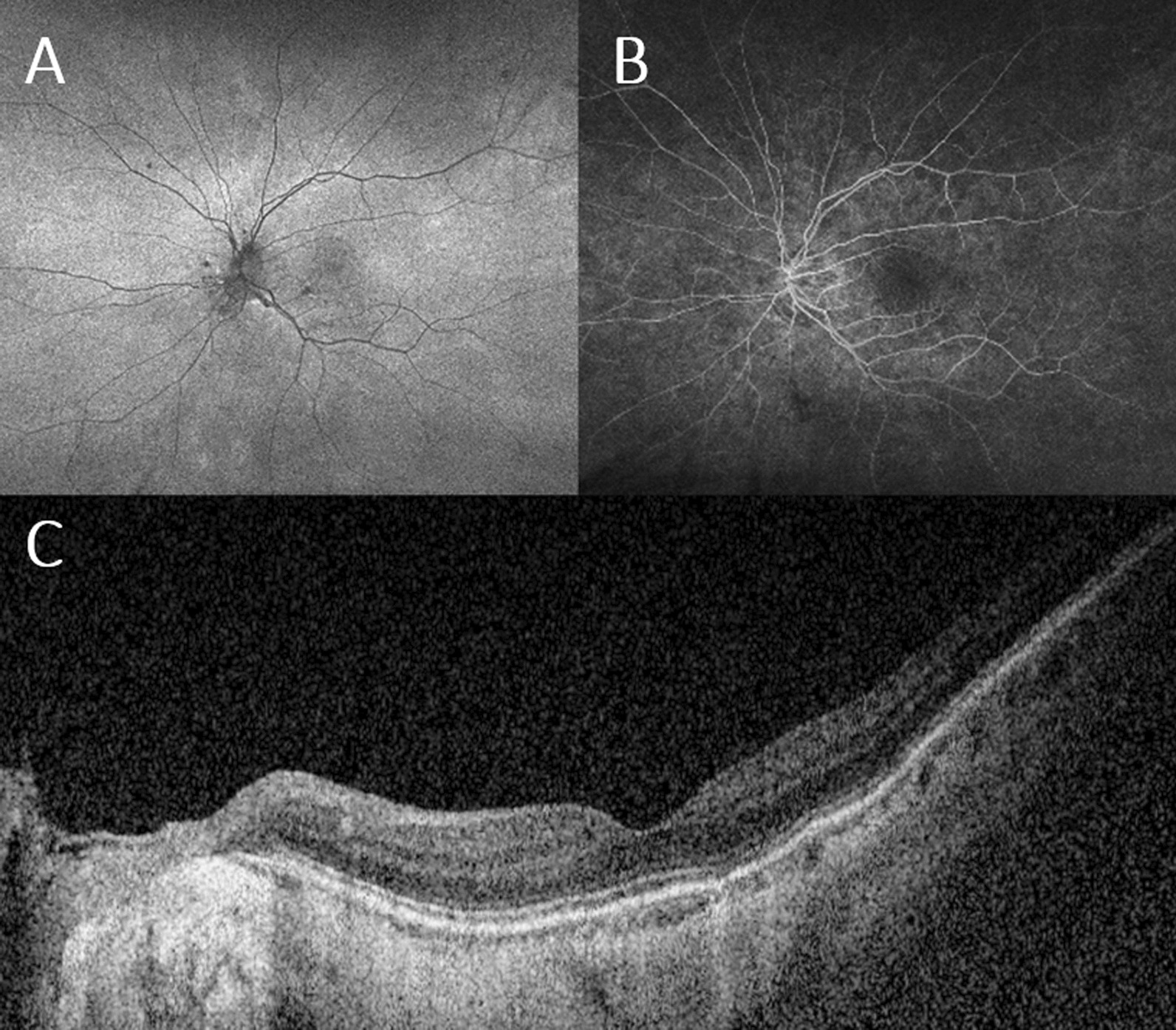
Multimodal imaging at Follow-up. Fundus autofluorescence (FAF) at one-month follow-u with significant improvement in peripapillary and macular hyper-autofluorescence. Fluorescein angiography (FA) at a 3-month follow-up shows resolution of wreath-like pattern hyperfluorescence in the 2-min frame. Enhanced depth imaging optical coherence tomography (EDI OCT) at presentation showing improvement of hyperreflective subretinal deposits and integrity of ellipsoid zone

We determined that the patient’s constellation of symptoms and characteristic imaging findings were consistent with MEWDS. The patient was prescribed a tapering course of oral steroids (Prednisone 40 mg daily for a week with a 10 mg weekly taper). At a 10-day follow-up from the presentation, the patient had no adverse events. The photopsia improved, and she had a visual acuity of 20/70 in the left eye. Hyperreflective subretinal deposits on OCT were reduced in size and number, and vitreous inflammation slightly improved. One month after the presentation, eye visual acuity improved to 20/50 and remained stable at the three-month follow-up visit. FAF and FA showed a significant improvement in peripapillary and macular autofluorescent spots and hyperfluorescent patterns, respectively (Fig. 2A, B). The ellipsoid zone was restored, and hyper-reflective deposits were resolved on OCT (Fig. 2C).

## Discussion

We presented a 69-year-old myopic female patient who developed MEWDS following a COVID-19 infection. Differentials for MEWDS include other white dot syndromes, mainly acute zonal occult outer retinopathy (AZOOR) and acute macular neuroretinopathy (AMN). AZOOR and AMN were less probable diagnoses, given the resolution of the ocular symptoms and improved imaging findings. Vitreoretinal lymphoma was also considered as a possible differential diagnosis but ruled out given the rapid resolution of symptoms and posterior segment findings and response to oral steroids.

COVID-19 infection has been associated with various retinal and uveal disorders, typically weeks after onset of COVID-19 symptoms [9]. There are not many reported MEWDS cases post-COVID-19 infection, though there are multiple reported cases post-COVID-19 vaccination [10]. The mechanism of action of COVID-19 in the pathogenesis of ocular disorders such as MEWDS is still being debated. One proposed mechanism of action is through angiotensin-converting enzyme 2 targeting SARS-CoV-2 on vascular endothelial cells, leading to impeded blood barrier function, disturbance of intercellular junctions, and cellular swelling [11]. Studies suggest that patients' retinal arteries and veins post-COVID infection sustain microvascular damage compared to unexposed patients [12, 13]. Disruptions in retinal peripapillary circulation are thought to contribute to the development of MEWDS [11]. Although the pathogenesis is still highly debated and relatively unknown, there are some associations of MEWDS with the HLA-B51 subtype. [14]

MEWDS is typically managed with observation as it is self-resolving. Oral corticosteroids can be considered if significant visual impairment is prolonged [15]. Recurrence of MEWDS is uncommon at a rate of about 14%, and most cases resolve spontaneously within a few weeks [1]. We started oral steroids for our patient, given her symptoms had been existing for ten weeks when she attended our clinic. Although the ocular symptoms and exam findings showed a significant improvement ten days after the start of oral steroid, it might have happened without this treatment, given the benign course of this disease.

The oldest patient featured on a case report of COVID-19 infection and MEWDS association was a 47-year-old female, and the oldest with COVID-19 vaccination and MEWDS association was a 67-year-old female. [5, 16]. Our case features a patient outside the typical age range of 20–40 years but follows the expected unilateral manifestation. To our knowledge, this is the oldest patient with a reported case of MEWDS associated with COVID-19 infection.

## Conclusion

MEWDS is a rare white dot syndrome that might be associated with both COVID-19 vaccination and infection. This is one of many ophthalmic disorders reported following COVID-19 infection.

## Data Availability

All data generated or analyzed during this study are included in this published article.

## Abbreviations

MEWDS: Multiple evanescent white dot syndrome
COVID-19: Coronavirus disease 19
PVD: Posterior vitreous detachment
OCT: Optical coherence tomography
FAF: Fundus autofluorescence
FA: Fluorescein angiography
RAPD: Relative afferent pupillary defect
EZ: Ellipsoid zone
CBC: Complete blood count
CMP: Complete metabolic panel
QFT: QuantiFERON-TB Gold
ACE: Angiotensin-converting enzyme
ANCA: Antineutrophil cytoplasmic antibodies
RPR: Rapid plasma reagin
FTA-ABS: Fluorescent treponemal antibody absorption
CRP: C-reactive protein
ESR: Erythrocyte sedimentation rate
AZOOR: Acute zonal occult outer retinopathy
AMN: Acute macular neuroretinopathy
